# Fatal pulmonary hemorrhage, pneumothorax and skin necrosis caused by IRIS to an *Aspergillus flavus* infection in a young patient with metamizole associated agranulocytosis

**DOI:** 10.1007/s15010-023-02149-x

**Published:** 2023-12-22

**Authors:** Micha Banz, Andreas Stallmach, Nikolaus Gaßler, P. Christian Schulze, Michael Fritzenwanger, Oliver Cornely, Oliver Kurzai, Mathias W. Pletz

**Affiliations:** 1 Institute of Infectious Diseases and Infection Control, Jena University Hospital, Jena, Germany; 2 Department of Gastroenterology, Hepatology and Infectology, Jena University Hospital, Jena, Germany; 3 Institute of Pathology, Jena University Hospital, Jena, Germany; 4 Department of Cardiology, Angiology, and Pulmonology, Jena University Hospital, Jena, Germany; 5 Department I of Internal Medicine, Faculty of Medicine and University Hospital Cologne, Excellence Center for Medical Mycology, Cologne, Germany; 6 Institute for Hygiene and Microbiology, University of Würzburg, Würzburg, Germany

**Keywords:** Immune reconstitution inflammatory syndrome (IRIS), Hemophagocytic lymphohistiocytosis (HLH), Ulcerative colitis, *Aspergillus flavus*, Invasive fungal disease, Immunosuppression

## Abstract

We report the case of a young female with steroid-dependent ulcerative colitis (UC) who developed a complex systemic infection with *Aspergillus flavus*. This occurred following a UC relapse while vacationing in the Middle East, leading to extended use of metamizole and subsequent agranulocytosis. On her return to Germany, she was hospitalized for neutropenic sepsis and later transferred to our hospital due to persistent cytopenia and suspected Hemophagocytic Lymphohistiocytosis (HLH). Despite initial stabilization with targeted treatment for pulmonary *Aspergillus flavus* infection, her condition rapidly deteriorated following the onset of an Immune Reconstitution Inflammatory Syndrome (IRIS), which manifested as skin necrosis and pneumothorax after the replenishment of neutrophil granulocytes. The patient eventually died from an unmanageable pulmonary hemorrhage. Microscopy of skin necroses showed a massive presence of *Aspergillus flavus*, but tissue culture remained negative, suggesting effective antifungal treatment yet delayed phagocytosis due to agranulocytosis. This case underscores the need to consider IRIS in immunosuppressed patients who worsen despite aggressive and appropriately targeted treatment, highlighting its potential beyond the commonly recognized context in HIV-positive patients.

## Case presentation

The patient, a 30-year-old female, had a long-standing history of UC since her initial diagnosis in January 2020. Even though she had been started early on a regimen of azathioprine and prednisolone, she has remained steroid-dependent, with repeated relapses during attempts to taper her dosage. During traveling to a Middle Eastern country, she experienced escalated symptoms, including mucous diarrhea up to sixteen times a day, fever, and occasional vomiting. She self-medicated with excessive amounts of metamizole (at least a 5 ml and a 10 ml bottle where used, adding up to 35 g of metamizole), leading to sporadic episodes of weakness and dizziness.

Upon her return to Germany, the patient was first admitted with dyspnea and fever to a regional hospital where she was initiated on empirical piperacillin/tazobactam, suspecting sepsis. Four days later, she was transferred to our facility with a provisional diagnosis of hemophagocytic lymphohistiocytosis (HLH). This diagnosis was confirmed on her meeting five of the eight diagnostic criteria: fever, splenomegaly, cytopenia affecting both platelets and neutrophils, elevated ferritin levels (peak 27.693 µg/l), and increased levels of soluble IL-2 receptor (peak > 750,000 U/ml). Treatment was started with 3 × 8 mg of dexamethasone. This initial diagnosis was later confirmed by a bone marrow examination.

**Fig.1:**
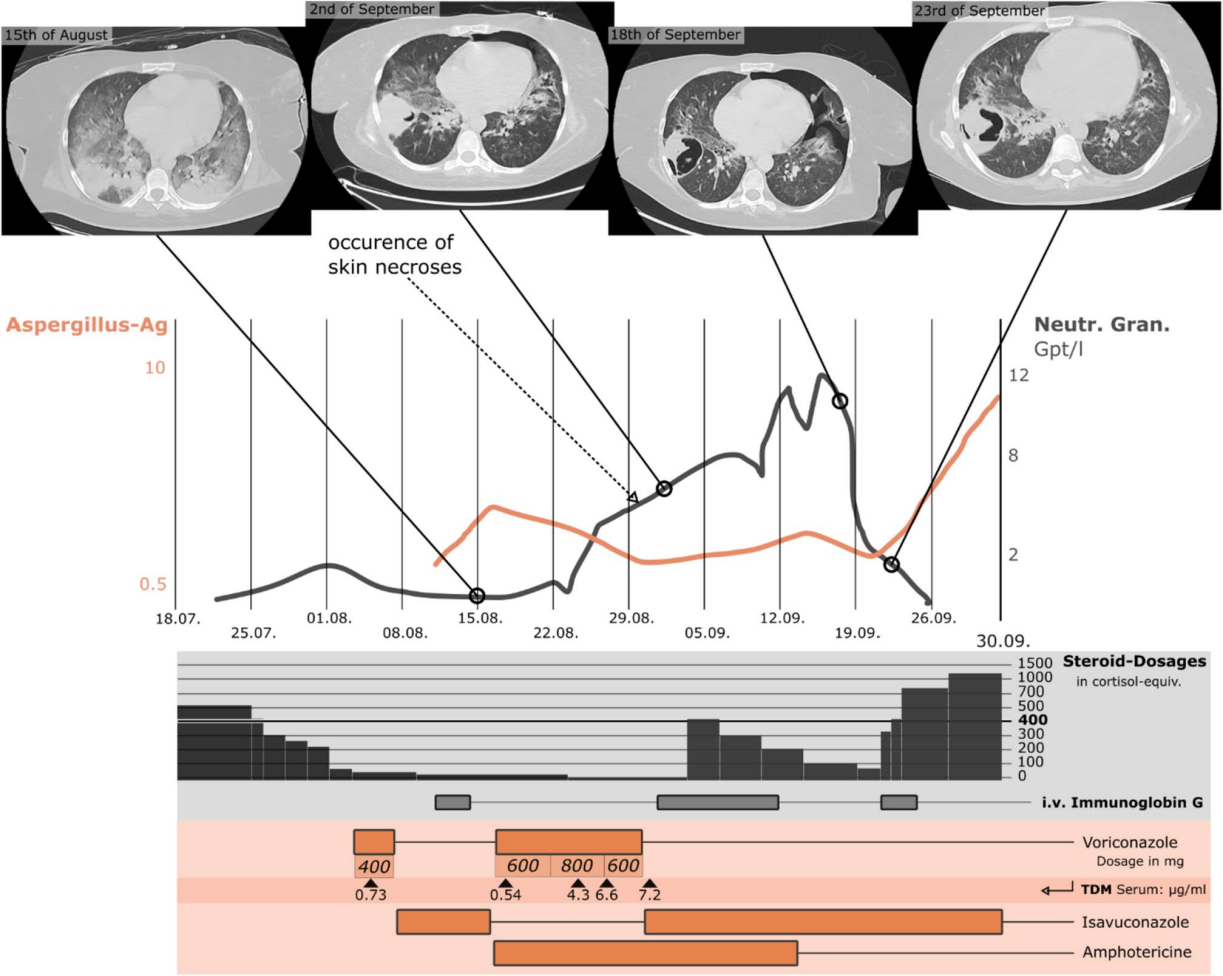
A timeline during the hospital stay displays Galactomannan (Aspergillus-Ag) levels indicating the extent of the fungal infection, and neutrophil granulocytes count (Neutr. Gran. in Gpt/l = gigaparticle per liter) reflecting immune recovery. Alongside neutrophil levels, select lung CT-Scan images highlight fungal progression and pneumothorax. The chart also illustrates the administration of glucocorticoids, depicted in terms of cortisol-equivalency or anti-inflammatory potency, with 400 being the therapeutic threshold for IRIS, and the administration of intra venous Immunoglobulin G and three antimycotic treatments targeting *Aspergillus flavus*. For the voriconazole dosage regimen, it is given in milligrams and administered twice daily, with therapeutic drug monitoring for serum levels. Isavuconazole is administered at 200 mg/day, and Amphotericin at 250 mg/day, both at consistent dosages

At our facility, the patient presented with fever and tachycardia, accompanied by abdominal swelling and diminished bowel sounds. By initial microbiological work-up of bronchoalveolar lavage *Aspergillus flavus* was identified by culture and consecutive mass spectrometry and cytomegalovirus (CMV) of 6.7 × 10^5^ copies/ml by PCR. *A. flavus* was later tested pan-sensitive using culture methods. Blood tests displayed a persistent cytopenia, a CRP level of 110 mg/dl, and a procalcitonin level of 2.8 ng/ml. A rectoscopy exposed severe UC (Mayo 3). Sequential CT scans of the lungs highlighted severe fungal pneumonia as illustrated in Fig. 1. Additional radiological evaluations of abdominal CT scans revealed pancolitis, pronounced mesenteric lymphadenopathy, hepatosplenomegaly, and significant edema of the bowel wall. The patient’s condition declined, culminating in acute renal failure and the necessity for dialysis. In management of her clinical complications, the patient’s Prednisolone dosage was methodically reduced from 70 mg at admission to 2.5 mg per day at 6 weeks after admission, as depicted in Fig. 1. This modification was intended to optimize the patient’s immune response to tackle the *Aspergillu*s infection.

**Fig. 2:**
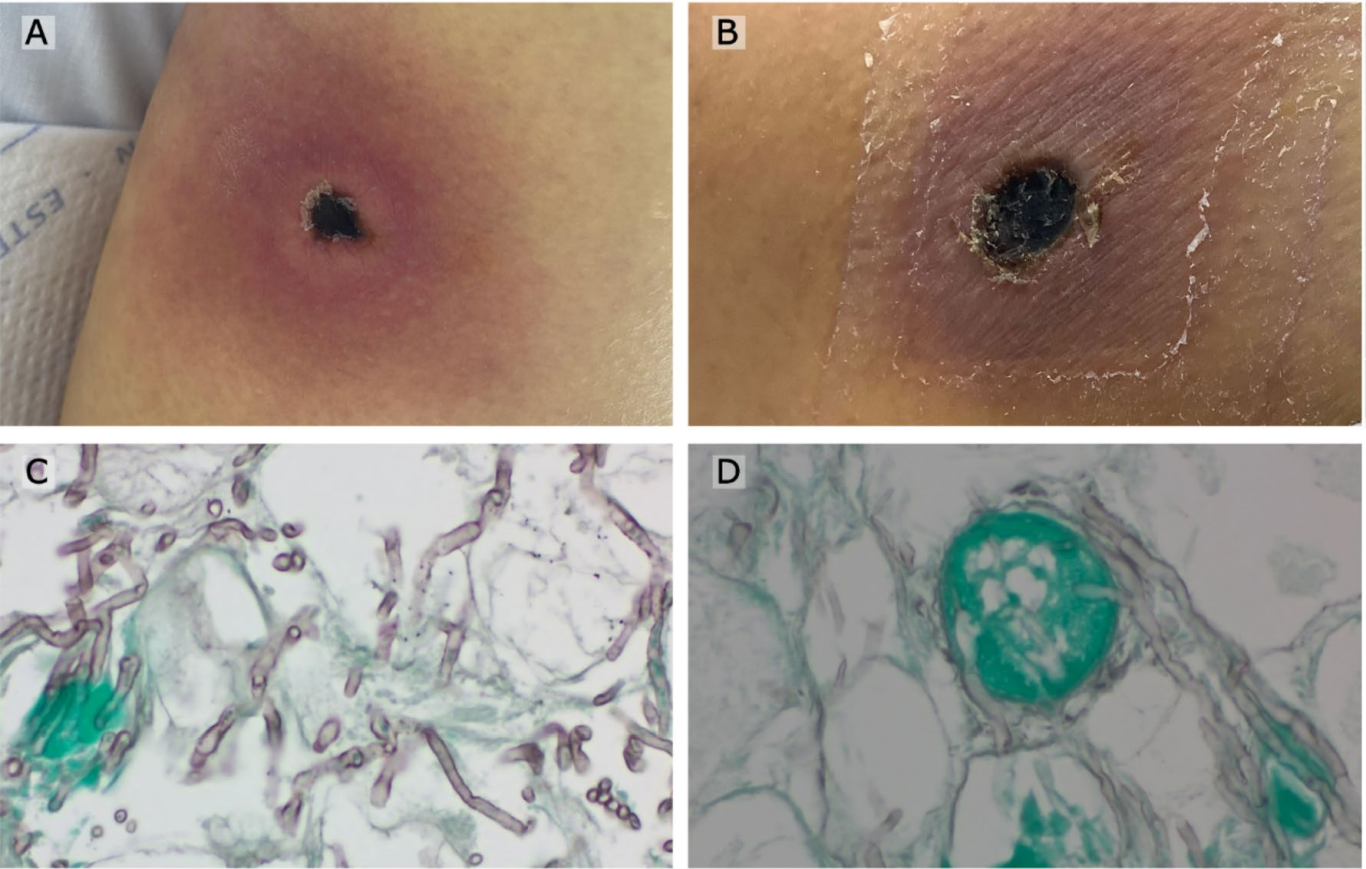
**A** and **B** Highlight granulomatous skin necroses. Pathological assessment of these areas using Grocott Gomori staining, revealed the mycelial network structures, depicted in microscopic views (**C** and **D)**

Six weeks after initial admission, multiple necrotic lesions manifested across various parts of her body (Fig. 2A, B). Histopathological evaluation of the skin lesions revealed pronounced necrosis of the fibrolipomatous tissue, with evidence of invasive mycosis and intermittent angioinvasive dissemination patterns (Fig. 2B, C). We interpret the presence of fungal elements in this context as probably non-viable pathogens remaining post effective antifungal treatment caused by the patient’s delayed immune clearance of fungal debris. However, it is important to consider, as highlighted by Donnelly et al. [1], that such an interpretation must be made cautiously, given the known limitations in the sensitivity of culture methods for detecting live fungal pathogens.

Along with these findings, the patient’s UC symptoms progressively intensified, making a subtotal colectomy with ileostomy essential by August’s end. This surgery was vital to address the continual bleeding, which was inducing recurrent anemia.

Two months after admission, despite the aggressive antifungal and antibiotic treatments in accordance with medical guidelines the patient’s infectious symptoms worsened, in contrast to the declining Aspergillus-antigen levels. As the patient’s bone marrow recovered, blood tests revealed an increase in neutrophil granulocytes count, as depicted in Fig. 1. This surge in immune response was concurrent with a decline in the patient’s overall health and the onset of a spontaneous pneumothorax, which became more severe in the subsequent days, as displayed in Fig. 1. This clinical pattern, combined with observations suggesting the possibility of non-viable Aspergillus persisting in the affected sites, and the correlation with granulocyte recovery, fostered the initial suspicion of tissue damage attributable to an IRIS. In response, an increased dose of 150 mg/day of prednisolone was given and daily infusions of immunoglobulins was initiated, in line with current literature [2].

During the further course, the granulocyte count decreased again and the pneumothorax resolved. However, the detection of an abdominal abscess mandated further surgical intervention and the microbiological work-up of abscess content revealed *Saccharomyces cerevisiae*. Complicating matters further, liver dysfunction also emerged. The patient tragically suffered an extensive bronchial hemorrhage and multi-organ system failure, which most likely led to the deterioration resulting in the patient’s passing on September 30th, 3 months after initial admission. The relatives declined an autopsy.

## Discussion

Since introduction of ART for HIV-positive patients, IRIS related to a restoration of the adapted cellular immune system has been recognized and extensively studied. Classic ART-induced IRIS is tied to the recovery of CD4^+^ cells that were previously decimated by the HI-Virus. Roughly 4 to 6 weeks after initiating ART, some patients may face an intense immune reaction from these renewed T cells. Intended to fight opportunistic infections that are mainly attacked by T cells such as toxoplasmosis, cryptococcosis, tuberculosis etc., this can spiral into a ‘cytokine storm’ causing severe symptoms [3, 4].

However, little is known about IRIS to opportunistic infections that are usually associated with granulocyte rather than a T-cell response such as *Aspergillus* spp. According to Dellière et al. *Aspergillus*-related IRIS can occur during neutrophil recovery, particularly after chemotherapy for acute leukemia or in stem cell recipients [5, 6]. The study reports a cohort of 19 patients where the mean time to clinical and radiological findings of IRIS from an absolute neutrophil count > 100/µL and > 500/µL was 3.5 days and 2 days, respectively. In addition, a few cases of *Aspergillus*-related IRIS have been described in lung transplant recipients. Pulmonary hemorrhage is associated in pulmonary aspergillosis with a rapid recovery of neutrophils [7, 8]. In addition, it is reported that IRIS related to fungal infections are clinically recognized for their occurrence of granulomatous necrosis, especially in *Aspergillus* infections [9, 10]. Furthermore, Dellière et al. states that the use of a colony-stimulating factor appears to be associated with the occurrence of IRIS in patients with invasive pulmonary aspergillosis with neutropenia. The study also mentions a case report describing a severe exacerbation of chronic cavitary pulmonary aspergillosis (CCPA) after granulocyte colony-stimulating factor (G-CSF) administration. Studies like the CANPHARI trial (NCT01916057), emphasizing Candida-derived IRIS, will provide invaluable insights into the nuances of immune reconstitution by focusing on specific pathogens [11].

During the clinical course of our patient, manifestations such as necrotic skin lesions, which appeared initially concomitantly with a declining galactomannan-levels, as well as a pneumothorax, and pulmonary hemorrhage were observed. These manifestations have been documented in primary literature in the context of IRIS related to *Aspergillus* infections [10, 12]. However, each of the mentioned symptoms have been individually reported in smaller studies, predominantly among patients with malignancies and/or tuberculosis [10]. The predominant pathophysiological mechanism is often attributed to Chronic Pulmonary Aspergillosis (CPA) which patients might acquire during periods of immunosuppression induced by the therapeutic interventions. Regular corticosteroid intake, even at low doses, further exacerbates this susceptibility [7].

In retrospect, since an autopsy was refused, it is difficult to determine what the etiology of the final bronchial hemorrhage was. On the one hand, of course, the same mechanism that contributed to the development of the necrosis, an exuberant granulocyte reaction to fungal mycelia already killed by antimycotics but not yet cleared, may have led to an erosion of a bronchial vessel. However, it is also conceivable that it was a late consequence of the initial HLH. HLH apparently also results in increased vascular vulnerability in the medium term and a substantial proportion of patients who die of HLH die of a final hemorrhage [13]. A third option could be a resurgence of *Aspergillus* infection, as galactomannan increased again in the last days before death. However, it must be considered that galactomannan does not have a high specificity and can also rise during the infusion of antibodies and blood products, and that we could not detect fungal growth in the respiratory cultures taken in many cases [14]. Furthermore, it is not clear whether galactomannan is also released during the clearance of the fungal mycelium killed by antimycotics due to the excessive granulocyte function [15, 16].

The diagnostic complexity of this case was augmented by the concomitant presence of HLH which was diagnosed by meeting five of eight of the HLH-2004 criteria [17] and later in the course of hospitalization confirmed by bone marrow investigation. The pulmonary haemorrhage could also be a consequence of the HLH, since fatal bleedings have been described in HLH case series and case reports [18].

Although HLH has a distinct molecular pathophysiology, its clinical presentation often closely mirrors that of IRIS, making differentiation challenging. The secondary/acquired form of HLH primarily occurs in adults and is commonly associated with infections, malignancies, autoimmune diseases, and acquired immunodeficiency states. These states include conditions such as acquired immunodeficiency syndrome (AIDS), transplantation, chemotherapy, chronic steroid use or other forms of immunosuppressive treatment. Such associations position HLH as a potential foundation for IRIS [19, 20].

The interplay between HLH and IRIS is yet to be understood in completion, and it is unclear if one condition predisposes or triggers the other, as might have been the scenario in this patient’s case. Studies on the levels of basic science as well as clinical investigations need to be conducted in the future to make sense of the interplay between HLH and IRIS and further understand the dynamics of host reaction syndromes [20, 21].

## Conclusion

Clinicians should remain alert for the possibility of IRIS in patients undergoing granulocyte immune reconstitution, directing particular attention to cases beyond the typical HIV-positive patients receiving ART. While IRIS development in HIV is closely monitored with swift treatment interventions, it is usually not considered in other immunocompromised conditions and related opportunistic infections. The lack of specific diagnostic criteria and the lack of clinical studies make the diagnosis and management of IRIS outside HIV challenging. We suggest, however, that (i) the timely association of new onset pulmonary (resulting in a pneumothorax) and skin tissues necroses with the sudden restoration of granulocytes after (ii) several days or weeks of appropriate antifungal treatment (50 days in the presented case) and (iii) the discrepancy between abundant detection of an invasive microorganism in tissue samples that cannot be grown in culture should rise suspicion towards an *Aspergillus-related* IRIS.

## Data Availability

Due to data protection regulations and the patient’s passing, the data used in this study cannot be accessed or made available for external use. Requests for further information should be directed to the corresponding author. These declarations were made in accordance with the university’s ethics committee and data protection regulations.
